# Partial Volume Reduction by Interpolation with Reverse Diffusion

**DOI:** 10.1155/IJBI/2006/92092

**Published:** 2006-02-14

**Authors:** Olivier Salvado, Claudia M. Hillenbrand, David L. Wilson

**Affiliations:** ^1^ Department of Biomedical Engineering, Case Western Reserve University, , Cleveland, OH 44106, USA; ^2^ Department of Radiological Sciences, St. Jude Children's Research Hospital, Memphis, TN 38105, USA; ^3^ Department of Radiology, University Hospitals of Cleveland & Case Western Reserve University, Cleveland, OH 44106, USA

## Abstract

Many medical images suffer from the partial volume effect where a boundary between two structures of interest falls in the midst of a voxel giving a signal value that is a mixture of the two. We propose a method to restore the ideal boundary by splitting a voxel into subvoxels and reapportioning the signal into the subvoxels. Each voxel is divided by nearest neighbor interpolation. The gray level of each subvoxel is considered as “material” able to move between subvoxels but not between voxels. A partial differential equation is written to allow the material to flow towards the highest gradient direction, creating a “reverse” diffusion process. Flow is subject to constraints that tend to create step edges. Material is conserved in the process thereby conserving signal. The method proceeds until the flow decreases to a low value. To test the method, synthetic images were downsampled to simulate the partial volume artifact and restored. Corrected images were remarkably closer both visually and quantitatively to the original images than those obtained from common interpolation methods: on simulated data standard deviation of the errors were 3.8%, 6.6%, and 7.1% of the dynamic range for the proposed method, bicubic, and bilinear interpolation, respectively. The method was relatively insensitive to noise. On gray level, scanned text, MRI physical phantom, and brain images, restored images processed with the new method were visually much closer to high-resolution counterparts than those obtained with common interpolation methods.


Hindawi Publishing Corporation
International Journal of Biomedical Imaging
Volume 2006, Article ID 92092, Pages 1–13
DOI 10.1155/IJBI/2006/92092

### Partial Volume Reduction by Interpolation with Reverse Diffusion

**Olivier Salvado,[1] Claudia M. Hillenbrand,[2] and David L. Wilson[1,3]**

[1] *Department of Biomedical Engineering, Case Western Reserve University, Cleveland, OH 44106, USA*
[2] *Department of Radiological Sciences, St. Jude Children's Research Hospital, Memphis, TN 38105, USA*
[3] *Department of Radiology, University Hospitals of Cleveland & Case Western Reserve University, Cleveland, OH 44106, USA*

Received 16 August 2005; Revised 26 November 2005; Accepted 27 November 2005

Recommended for Publication by Guowei Wei

Many medical images suffer from the partial volume effect where a boundary between two structures of interest falls in the midst of a voxel giving a signal value that is a mixture of the two. We propose a method to restore the ideal boundary by splitting a voxel into subvoxels and reapportioning the signal into the subvoxels. Each voxel is divided by nearest neighbor interpolation. The gray level of each subvoxel is considered as "material" able to move between subvoxels but not between voxels. A partial differential equation is written to allow the material to flow towards the highest gradient direction, creating a "reverse" diffusion process. Flow is subject to constraints that tend to create step edges. Material is conserved in the process thereby conserving signal. The method proceeds until the flow decreases to a low value. To test the method, synthetic images were downsampled to simulate the partial volume artifact and restored. Corrected images were remarkably closer both visually and quantitatively to the original images than those obtained from common interpolation methods: on simulated data standard deviation of the errors were 3.8%, 6.6%, and 7.1% of the dynamic range for the proposed method, bicubic, and bilinear interpolation, respectively. The method was relatively insensitive to noise. On gray level, scanned text, MRI physical phantom, and brain images, restored images processed with the new method were visually much closer to high-resolution counterparts than those obtained with common interpolation methods.

Copyright © 2006 Olivier Salvado et al. This is an open access article distributed under the Creative Commons Attribution License, which permits unrestricted use, distribution, and reproduction in any medium, provided the original work is properly cited.

### 1. INTRODUCTION

We have developed a 2D method for image interpolation that uses reverse diffusion to correct for the partial volume effect. Medical images almost always suffer from a partial volume effect where the boundary between two structures of interest falls in the midst of a voxel leading to blurred voxels having inaccurate intensities. When a digital image is acquired, signal at frequencies greater than half the sampling frequency is aliased or lost if antialiasing filter is used. The acquired image contains only low frequencies which introduces blurring of edges. The problem we are facing is then to recover the high frequencies from the edges, without changing the images where information from finer details has been irremediably lost by the acquisition process. We model the signal of a voxel as the integral of the signal over the surface delimited by the voxel limits. We propose a method to interpolate while recovering the ideal boundary by splitting a voxel into subvoxels and reapportioning the subvoxels' signal. We designed this method to correct magnetic resonance imaging (MRI) scans where partial volume is a considerable limitation, but many other digital imaging applications would benefit from more accurate image intensity. For example, this is also applicable to x-ray imaging where there is a partial area effect, digital photography such as satellite imaging, as well as text scanning. The method is applied to 2D data but extensions to 3D should be possible. Here we keep our discussion to 2D but use the term voxel because most medical images have a thickness.

There are many methods for image interpolation and commonly used ones in medical field include nearest neighbor, bilinear, and bicubic spline. To optimally reconstruct a continuous band-limited signal, one can apply sinc interpolation in the spatial or frequency domains to samples acquired with consideration to the sampling theorem [1]. Various approximations to the infinite sinc function in the spatial domain are proposed that address the tradeoff between blurring and ringing artifacts [2, 3]. Such interpolation methods use only the gray-level information and make no assumption about the partial volume process. However, the structures that we wish to image are seldom band limited (e.g., the air/skin interface is a step edge having an infinite bandwidth). More advanced schemes have been designed such as shape interpolation based upon binary or gray-scale image data [4]. Other methods use nonlinear schemes to enhance edges for increased visual quality, but rarely rely on physical modeling of the imaging process [5–7]. In particular, diffusion-based methods have been proposed to achieve super-resolution. Most of those techniques start from a blurred interpolated images (e.g., using bicubic interpolation) and sharpen edges by controlling the diffusion such that homogenous regions are filtered and edges between them are enhanced [8–11]. Those techniques achieve impressive visual quality enhancement but they may generate extra features, which may be the reason that they are seldom used for interpolating medical images. In this new algorithm, we seek to reduce partial volume effect in medical images without creating artifact, and with no consideration to visual image quality.

Classification methods exist that enable partial volume correction of areas and volumes. These methods model the image histogram, often as a Gaussian mixture, and use spatial information between voxels to estimate the mix inside each voxel of the different classes [12–16]. Almost all of these methods assume that a voxel can contain at most two tissue types. The performance of these methods depends upon the accuracy of histogram modeling and often specific parameters such as the number of classes. Such methods can be used to perform measurements and create high-resolution labeled images. As far as we know, these methods have not been extended to gray-scale image interpolation.

Below, we describe our algorithm (Section 2) and the experimental methods for evaluating the algorithm using synthetic images and MR images of a physical phantom and human brain (Section 3). Results from the proposed method are compared to conventional interpolation methods in Section 4 followed by a discussion and conclusion.

### 2. ALGORITHM

### 2.1. Assumptions and image model

We assume that the original data present sharp steps between homogenous regions and that partial volumes/areas result in a blurring of edges. Further, we assume a model for the voxel aperture where the signal in a voxel is the integral of the signal over the volume delimited by the voxel. That is, the partial volume/area imaging process conserves signal. In medical images, the assumption of step edges is clearly relevant at the interface between tissues such as bones/muscles, vessel wall/lumen, and skin/air. Other tissue interfaces might not present step edges such as the edge of a tumor with invaginations into normal tissue. Nevertheless, the assumption is valid whenever transitions are "sharper" than the voxel size, which is often the case because experience shows that higher-resolution clinical MR images typically result in sharper edges. We assume also 8-neighbor connectivity of homogeneous regions and that the point spread function of the imaging apparatus is not bigger than the voxel size.

A brief description of the proposed method follows. It does not rely on classification and histogram modeling. Subvoxels are created using nearest neighbor interpolation, and the gray level of each subvoxel is considered as "material" able to move between subvoxels. A partial differential equation is written to allow the material to flow towards the highest gradient direction, creating a "reverse" diffusion (RD) process. Changing the sign of the time variable leads to a highly unstable equation. Attempts to numerically solve this ill-posed problem have relied on regularization of the numerical solution by minimizing a functional [17–19], or approximation of the near past solution from the estimation of well-posed forward values [20]. The original anisotropic diffusion scheme [21] has been extended: Gilboa et al. [8] balanced forward and backward flows and Pollack et al. [9] merged regions to avoid instabilities generated by a discontinuity of the diffusion coefficient. These related methods do not include constraints to address the partial volume effect, and require specifying extra parameters. Some methods based on stabilized shock filters [22], and more recently, work by Breuss et al. [23] are also related to our algorithm. We present here some simple constraints that control the reverse diffusion in the context of image processing without any new free parameters. Moreover, convergence is straightforward and there is no need to specify a number of iterations. Flow is subject to constraints that tend to create step edges between regions of different intensities. Material is conserved in the process thereby conserving MR signal. It should lead to better results than pure interpolation methods since it uses more information, while avoiding the limitations of the pattern recognition techniques.

### 2.2. Constrained reverse diffusion

The method is illustrated in Figure 1 in 1D where we use the term "bin" instead of voxel. The original signal (a) is discretized causing a partial area/volume artifact (b). In (c), the discrete signal is oversampled into four identical subbins. Each gray value of the subbins is then increased or decreased (arrows) so as to restore the original signal (d). To determine the direction of the arrows in panel (c) each subbin is considered made of material that can either flow *to* a neighbor subbin if it has higher value or *from* it if it has a lower value. We implement this method iteratively.

Equations describing the flow process are now described on the one-dimensional example from Figure 1. The observed discrete signal subject to partial volume effect is $\tilde{y}_l$ with $l$ the original bin number out of $L$ total bins (Figure 1(b)). The over sampled signal is $y_i$ (Figure 1(c)), with $i$ the new binning out of $I$ bins ($I = 4L$ in this case). For a given subbin $i$, the amount of material flowing to, or from, the right side is $y_{i+1} - y_i$ and $y_{i-1} - y_i$ for the left side. Material will be lost if the flow is positive. The balance of material for a given subbin is the sum of the two sides, expressed mathematically as the divergence. Using an iterative approach the

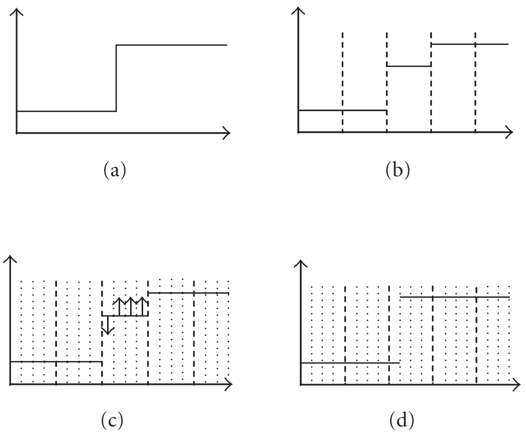

FIGURE 1: Principle of partial volume effect correction in one dimension. The original 2-class continuous ideal signal (a) is discretized as in (b) causing some bins to take an arbitrary value when multiple classes are present. After subsampling 4 times in (c) each subbins value is increased or decreased (arrows) to reach its correct value (d).

value $y_i$ can be computed at the iteration $t+1$ by

$$y_i^{t+1} = y_i^t - a\left[\left(y_{i+1}^t - y_i^t\right) + \left(y_{i-1}^t - y_i^t\right)\right] \tag{1}$$

with the constant $a > 0$ adapting the speed of the flow. This equation can be rearranged by extracting from $a$ the spatial size of the subbins $di$ and a time step $dt$ to yield a partial differential equation when $di$ and $dt$ tend to arbitrarily small values:

$$\frac{dy_i}{dt} = a' \frac{d^2 y_i}{di^2}. \tag{2}$$

If the constant $a'$ were negative, this last equation would be the diffusion or heat conduction equation. In this “reverse diffusion” case, the positive sign leads to a well-known unstable behavior. We next describe how to constrain the flows in a manner consistent with the assumptions described above.

To continue our analogy, in order for any material to leave a subbin, there must be enough material present. Since the “low level” of $y_i$ is not known, we approximate it using the minimum value of its neighbors. In addition, material can leave a subbin only if there is sufficient room to receive it. That is, the recipient subbin has not reached its “high level,” as approximated by the maximal value of *its* neighbors. Hence, the stability of the numerical scheme is enforced by limiting flow such that a subbin does not give or receive material such that it will contain material below its low level or above its high level, respectively. In 1D, each subbin can receive/give materials from/to two neighbors. We thus divide the possible flow by 2, equivalent to the CFL bound [24]. The constrained implementation, with $N_i$ the neighbor subbins of $i$, becomes

$$Q_i^{\max} = \frac{\max_{i' \in N_i}\left(y_{i'}\right) - y_i}{2}; \text{ maximal outward flow,} \tag{3}$$

$$Q_i^{\min} = \frac{y_i - \min_{i' \in N_i}\left(y_{i'}\right)}{2}; \text{ maximal inward flow,} \tag{4}$$

$$Q_{i,i+1} = \max\left[-Q_i^{\max}, -Q_{i+1}^{\min}, \min\left(Q_{i+1}^{\max}, Q_i^{\min}, y_{i+1}^t - y_i^t\right)\right]; \text{ total flow between subbins } i \text{ and } i+1, \tag{5}$$

$$y_i^{t+1} = y_i^t - Q_{i,i+1}, \tag{6}$$

$$y_{i+1}^{t+1} = y_{i+1}^t + Q_{i,i+1}. \tag{7}$$

Equation (5) reflects the material constraints from the neighbors. If $y_i < y_{i+1}$, material should move from bin $i$ to bin $i+1$, as determined by the positive gradient $y_{i+1} - y_i$, but this flow should be limited by the room left in bin $i+1$ ($Q_{i+1}^{\max}$) and the low level for bin $i$ ($Q_i^{\min}$). Therefore the total flow between $i$ and $i+1$ is given by $\min(Q_{i+1}^{\max}, Q_i^{\min}, y_{i+1}^t - y_i^t)$. Since $Q_i^{\max}$ and $Q_i^{\min}$ are always positive or null, the remaining terms in (5) constrain the flow when $y_i > y_{i+1}$.

### *2.3. Application to images*

For the 2D case, let $\widetilde{Y} = \{\widetilde{y}_{lm},\ l = 1,2,\ldots,L,\ m = 1,2,\ldots,M\}$ be the original image of size $L \times M$ voxels. Let $Y = \{y_{ij},\ i = 1,2,\ldots,I,\ j = 1,2,\ldots,J\}$ be the new image at the higher resolution, where every voxel has been divided into $R^2$ voxels ($I = RL$ and $J = RM$). In the 2D case, there are 2 flows to compute and distribute accordingly: horizontal and vertical. However the computation of $Q^{\min}$ and $Q^{\max}$ needs to be adapted. In both cases, the neighboring set considered is the eight-connected voxels of the recipient voxel $N_{ij}$. Since edges are favored, the “high level” is not the maximum from the set, but the 6th highest to ensure that at least 3-connected voxels are considered for an edge. Similarly, the 4th highest value defines the low level. Our first implementation using the maximal and the minimal values (9th and 1st instead of 6th and 4th) over the neighboring set $N_{ij}$ yielded artifacts for high and low levels were driven by noise and not homogeneous regions of the image. After careful analysis and experimentations, we believe that the scheme using the 6th and 4th rank-order statistic is the one giving the best results both quantitative and qualitative. This scheme can accommodate any edge configuration in $N_{ij}$. The same argument for numerical stability as above leads to dividing the flow limits by 4. The limits on the flows for a given voxel $ij$ are

$$Q_{ij}^{\max} = \frac{\operatorname{ord}_{\{i',j'\}\in N_{ij}}\left(6, y_{i'j'}\right) - y_{ij}}{4}, \quad Q_{ij}^{\min} = \frac{y_{ij} - \operatorname{ord}_{\{i',j'\}\in N_{ij}}\left(4, y_{i'j'}\right)}{4}, \tag{8}$$

where $\operatorname{ord}_{\{i',j'\}\in N_{ij}}(n, y_{i'j'})$ denotes the *nth* highest value over the set $N_{ij}$, see Figure 2 for illustration.

As described, the computation of the gradient is done at the subresolution level *(di,dj)* and can induce spatial cluster of materials: materials tend to aggregate into small islands for the flow might be driven by the noise gradient. To avoid this

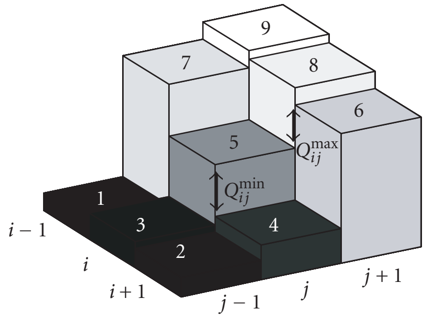

Figure 2: Drawing illustrating the computation of $Q^{min}$ and $Q^{max}$ for a voxel $ij$, using rank-order statistic. Numbers show the rank of each voxel gray level in this 3 × 3 neighborhood system; the lower the number, the smaller the intensity.

problem the gradient is computed on the filtered image such that materials flow in the direction of the brightest area of the image avoiding spatial clusters induced by local gradient fluctuation [25]. We use a Gaussian kernel whose standard deviation is half a voxel at the original resolution:

$$\hat{Y} = Y \otimes G(0, R/2), \tag{9}$$

where $G(0, \sigma)$ is a Gaussian kernel centered at the origin with $\sigma$ standard deviation. The equations for the horizontal and vertical flows are

$$\begin{aligned} Q_i &= \max\left[-Q_{ij}^{max}, -Q_{i+1j}^{min}, \min\left(Q_{i+1j}^{max}, Q_{ij}^{min}, \hat{y}_{i+1j}^{t} - \hat{y}_{ij}^{t}\right)\right], \\ Q_j &= \max\left[-Q_{ij}^{max}, -Q_{ij+1}^{min}, \min\left(Q_{ij+1}^{max}, Q_{ij}^{min}, \hat{y}_{ij+1}^{t} - \hat{y}_{ij}^{t}\right)\right]. \end{aligned} \tag{10}$$

The new image can now be updated taking into account the signal conservation constraint. That is, the signal in a voxel at the coarse resolution is the sum of the signals in the voxels at a higher resolution. A solution is to block any flow across pseudo-boundaries defined by the original resolution. Using this constraint, the signal will be reapportioned within an original voxel while preserving the sum over the subvoxels constant:

$$\begin{aligned} y_{ij}^{t+1} &= y_{ij}^{t} - B_{ij}\left(Q_i + Q_j\right), \\ y_{i+1j}^{t+1} &= y_{i+1j}^{t} + B_{ij}Q_i, \\ y_{ij+1}^{t+1} &= y_{ij+1}^{t} + B_{ij}Q_j, \end{aligned} \tag{11}$$

where $B_{ij}$ =0 when $i$ modulo $R$ =0 or $j$ modulo $R$ =0, and $B_{ij}$=1 otherwise.

The method consists of iterating the set of equations (8) through (11) in this order, and to apply it to all voxels (without identifying those lying on the edges). Typical behavior for the sum of the absolute flow is shown in Figure 3 top right: it decreases and converges to zero. To stop iterating, we chose to test if its value falls below a fraction of its maximum (< 0.1%). We tested also to stop when entropy was changing little as shown in Figure 3 bottom right. Entropy is physically appealing because it relates directly to the quantity of information present in the image. Correcting the partial volume effect is tantamount to reorganizing the information of the image in a way that is closer to the "true" image. Therefore, the reduction of the disorder should lead to a lower value of the entropy. Indeed when images are corrected one can observe a reduction of the entropy (Figure 3 bottom-right graph). When the algorithm converges, the entropy should stabilize to a low value, which could be tested to stop iterating the above equations. However, we found the entropy measure noisier than the total flow, and we use the latter as a stopping criterion.

### 3. EXPERIMENTAL METHODS

To evaluate the performance of the proposed method, we used a multiclass synthetic image $I_{true}$. Partial volume effect was simulated by averaging every four voxels into one voxel ($I_{red}$). The new image was then corrected ($I_{cor}$) and compared to the original. Since the original is known, several metrics can be used to quantify the result. We computed common criteria used in the literature to qualify interpolation algorithms [26]. We used the mean square difference (MSD),

$$\text{MSD} = \frac{\sum_{\Omega}\left(I_{cor} - I_{true}\right)^2}{\sum_{\Omega} 1}, \tag{12}$$

where $\Omega$ is the image domain, and the number of site disagreements (NSD) using a threshold value of 10% of the dynamic range:

$$\text{NSD} = \frac{100 \times \sum_{\Omega} \delta\left(\left|I_{cor} - I_{true}\right| > \text{threshold}\right)}{\sum_{\Omega} 1}, \tag{13}$$

where $\delta(c)$ is equal to one if the condition $c$ is true, and zero otherwise. In order to provide more meaningful values, we used the square root of the MSD divided by the dynamic range of the data. That is, the standard deviation expressed in percentage of the gray-level range. We refer to that error as the relative error (RE).

Several digital test images were created. First, a 192 × 256 *synthethic* image was generated with three tissue classes having gray values of 50, 100, and 150. Features in the image were created to mimic tissues interfaces. Outside and four disks inside the tissue, voxels were set at a low value of 20 to simulate air, and thus multiple tissue/air boundaries.

Second, we used the classic Lena image. We performed also some experiment on images where the original cannot be found but only approximated; therefore, the metrics defined above cannot be computed. We compared results of the proposed reverse diffusion method, to bicubic or linear interpolation as implemented in Matlab (MathWorks, Natick, Mass, USA).

Third, since our main interest is to correct for partial volume effect present in MRI, we conducted several experiments. MRI acquisition was performed on a Siemens Sonata 1.5 T scanner (Siemens, Erlangen, Germany). First, a phantom made of multiple tubes filled with four different solutions of agar gel [27] has been acquired with a T2W sequence and phased array surface coils. Imaging parameters for the turbo spin echo sequence (TR/TE/TI/NSA/thickness/

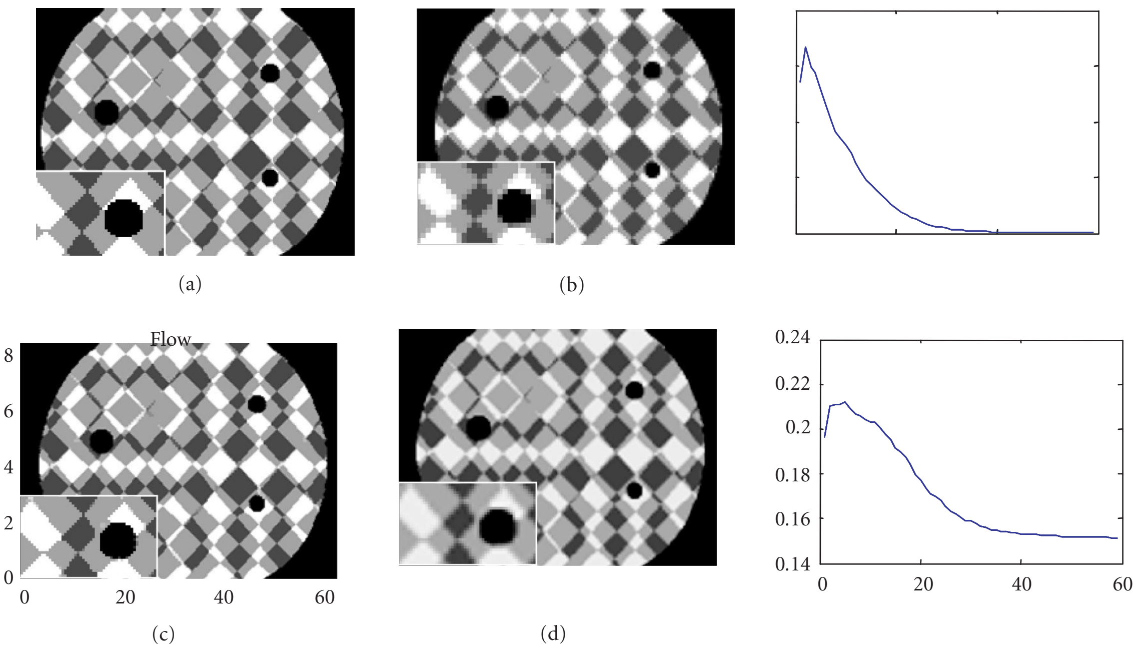

Figure 3: Test on synthetic image. An original 192 × 256 gray-level image (a) made of three classes plus background has been degraded as in (b) to simulate the partial volume effect. After 59 iterations (about 4.5-second CPU time) the corrected image is shown in panel (c) along with bicubic interpolation (d) of the low-resolution image. On the right side, the total flow over the image (top) as well as the entropy (bottom) is shown as a function of the iteration number. Images (c) and (d) are to be compared to (a), the original high-resolution image. Insets in each image are a magnified version of the background circle in the upper-right background circle.

FOV) were as follows: 2R-R/68 ms/600 ms/2/3 mm/13 cm. Fat saturation was applied. The in-plane resolution was $0.51 \times 0.51\,\text{mm}^2$. The image was corrected and edges of the tubes could be analyzed for circularity and sharpness in the case of multiple gray-level contrasts and the presence of strong intensity inhomogeneity.

Experiments with noise were done by adding centered normal noise to the original image as measured by the percentage of the noise standard deviation relative to the dynamic range. Tests ranging from 0% to 14%. Noise was added before partial volume simulation because noise during MRI acquisition comes mainly from weak signal, either because the number of proton is low in case of high resolution, or because magnetization has been lost due to relaxation (T1, T2, or T2*). Noise added after partial volume simulation would mostly simulate noise in the MRI equipment known to be low.

To test the ability to recover data from MRI, we obtained MR images of a physical phantom at various resolutions and tested our ability to recreate a high-resolution version. A cylindrical phantom filled with doped water and containing multiple size plastic rods (diameters: 13 mm, 10 mm, 7.5 mm, 6 mm, 5 mm, 4 mm, 3 mm, 2.5 mm, 2 mm, 1.5 mm, and 1 mm) has been imaged using the same field of view, but multiple resolution matrices: 128 × 128, 256 × 256, 512 × 512, and 1024 × 1024. The different images present different resolutions: $2\times 2\,\text{mm}^2$, $1\times 1\,\text{mm}^2$, $0.5\times 0.5\,\text{mm}^2$, and $0.125\times 0.125\,\text{mm}^2$, respectively. We corrected low-resolution images and compared them to their corresponding high-resolution images. Imaging parameters (TR/TE/NSA/slice thickness/FOV) for the T1W spin echo sequence were as follows: 400 ms/12 ms/1/5 mm/25.6 cm. Because it is difficult to compare directly images acquired with different parameters at different time points, we compared the area of the phantom in different images. Since it is a 2-class data (air/rods with no signal, and doped water with strong signal), the cumulative sum of the histogram presented a sharp change around the gray value in between the dark voxels of the background and the bright voxel of the phantom. An ideal image histogram would have only two peaks, its integral would present an ideal step. Because of noise and partial volume effect the data present a smooth step. We estimated the area of the water in the image by counting the number of voxels in the middle of the step and taking into account the in-plane resolution.

We acquired human images for testing the algorithm. Sagittal slices of a human head were acquired using a T1W acquisition as for the phantom above. Qualitative comparisons are made between slices at different resolutions, since even a small subject motion will hinder quantitative comparison. Finally, we corrected transverse images of human neck that were acquired for evaluation of atherosclerosis. For this latter application, MR scans were conducted on the same scanner as before with a custom-built, phased array coil, with two coils on each side of the patient's neck. Dark blood images were obtained using ECG-triggered double inversion recovery (DIR) turbo spin echo sequences. Imaging parameters (TR/TE/TI/NSA/thickness/FOV) were as follows: T1W: 1R-R/7.1 ms/500 ms/2/3 mm/13 cm; PDW: 2R-R/7.1 ms/600 ms/2/3 mm/13 cm; T2W: 2R-R/68 ms/600 ms/

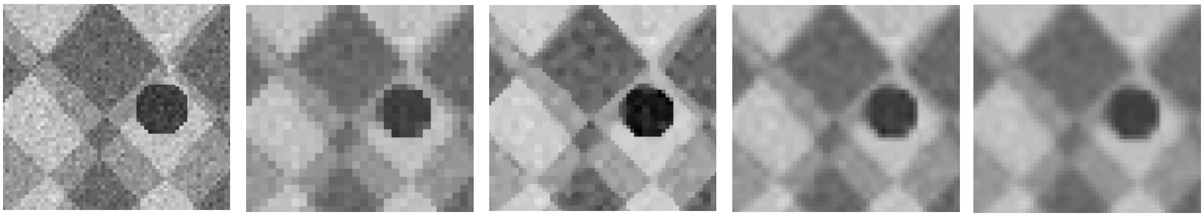

(a) (b) (c) (d) (e)

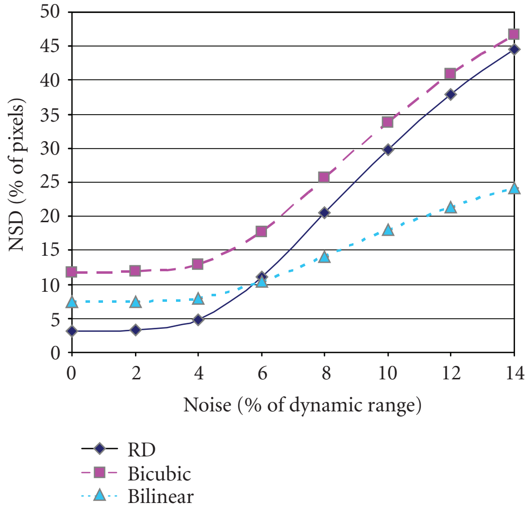

(f)

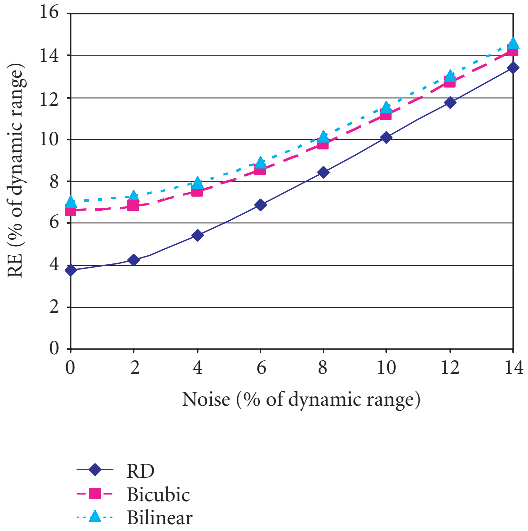

(g)

Figure 4: Performance on noisy synthetic image. Multiple level of normally centered noise, as measured as the percentage of standard deviation to dynamic range, has been added on the synthetic image from Figure 3 and corrected. Example of details is shown in the top row for 10% noise level: (a) original noisy image, (b) simulated partial volume effect, (c) corrected image with reverse diffusion, (d) bicubic interpolation, and (e) bicubic interpolation of (b). The two graphs show NSD (f) and RE (g) criteria for noise levels ranging from 0% to 14% (the lower the better).

2/3 mm/13 cm. Fat saturation was applied. The in-plane resolution was $0.51 \times 0.51\, mm^2$, and slice thickness was 3 mm.

### 4. RESULTS

### 4.1. Synthetic multiclass image

We evaluated our method on a multiclass synthetic image (Figure 3). The corrected image with the new algorithm is much closer to the high-resolution original than that interpolated with a bicubic kernel. Edges are sharp whereas bicubic interpolation introduces blurring. When three classes are mixed in a single voxel or when a detail is smaller than a voxel size (10 o'clock position on the border of the black circle zoomed in the insets), the algorithm cannot recover the original information reaching the Shannon information theory limit. In this example, the algorithm stopped after 59 iterations, about 4.5 seconds on a Pentium IV class personal computer, when the total flow over the image fell to less than 0.001 times its maximal value, as shown in the top-right graph. We implemented the algorithm in Matlab code on a Pentium IV 2.4 GHz personal computer. A more efficient implementation and a compilation step would certainly decrease the computing time.

Sensitivity to noise was tested on this synthetic image (Figure 4) with noise level ranging from 0% to 14% (noise standard deviation as a percentage of the dynamic range). The top row of pictures in Figure 4 shows details for the 10% noise-level case, with the algorithm stopped after 45 iterations. Visually RD gave crisp edges as compared to bicubic or linear interpolation indicating that partial volume is corrected even in the presence of noise. Quantitative measurements confirmed those results as shown in Figures 4(f) and 4(g), where the NSD and RE, respectively, are plotted. For the noise-free case the relative errors (RE), defined as the ratio of the standard deviation of the errors relative to the dynamic range, were 3.8%, 6.6%, and 7.1% for the proposed method, bicubic, and bilinear interpolation, respectively, and as compared to 7.2% before correction. As measured by RE, RD method is superior to bilinear and bicubic interpolations at all noise levels, but the improvement is reduced at the higher

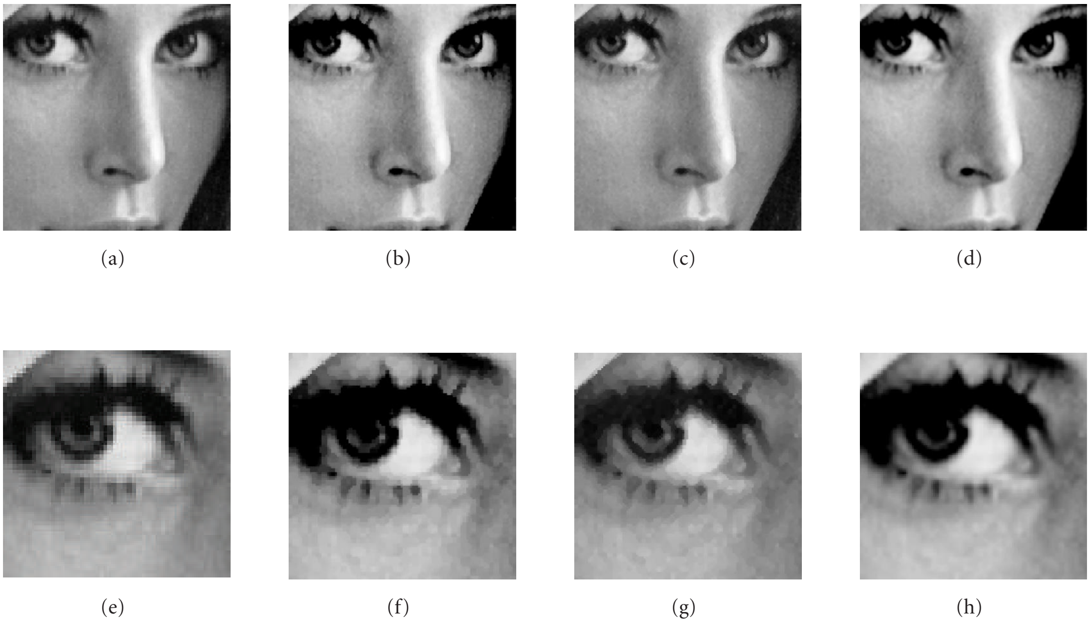

FIGURE 5: Original 100 × 100 voxels Lena picture (a) is interpolated with the proposed method two times (b) and four times (c) as well as four times with bicubic interpolation (d). Lower row of panels show a zoom on the left eye of the corresponding top row images. The interpolated image with RD ((c) and (g)) is much sharper than the bicubic interpolated one ((d) and (h)).

noise levels. For the NSD (Figure 4(e)), in the noise-free case, only 3.1% of the voxels have greater than 10% errors for RD, as compared to 11.7% for bicubic interpolation. As noise is increased, RD always outperforms bicubic. At high noise levels bilinear has a reduced NSD value, but this noise reduction effect comes at the expense of blurred edges. RD performs better than bicubic interpolation at all noise levels.

### 4.2. *Lena gray-level image*

Figure 5 shows result of the proposed method applied to the Lena image. The top row shows a cropped 100 × 100 detail, whereas the bottom row shows a zoom on the left eye. RD has been applied twice increasing the resolution by four. One can appreciate the reduction of the blocking artifact present in the original (a) and (e). The result images (c) and (g) are crisper than when interpolated with a bicubic kernel; for example, the lashes in the zoomed insets as well as the smoother contour of the nose and the chin. This technique might enhance digital artworks when the size of images needs to be increased.

### 4.3. *MRI physical phantom*

We imaged a MR phantom made of tubes to simulate gray-level images with different contrast, using surface array coils that induce important intensity inhomogeneity (Figure 6). Image resolution was increased four times and tube edges presented sharp boundaries, without the blurring associated with bicubic interpolation. No artifact from the intensity inhomogeneity was noted, and circular shapes became smooth as one should expect.

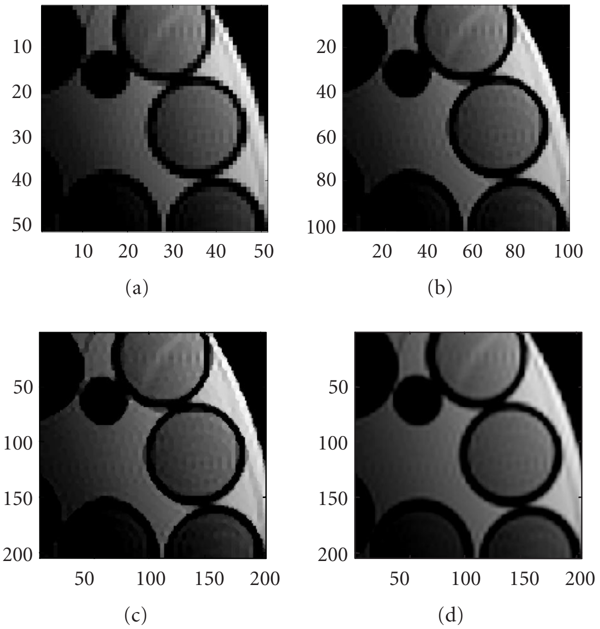

FIGURE 6: Correction of an actual MRI image of a physical phantom. The original T2W image is shown in panel (a) with an in-plane resolution of 0.72 × 0.72 mm. The proposed method is applied two times doubling (b) and quadrupling (c) the number of voxels. For comparison, the original image interpolating four times with bicubic interpolation is shown in panel (d).

A standard multiresolution phantom allowed us to compare the correction of structures of different size (Figure 7).

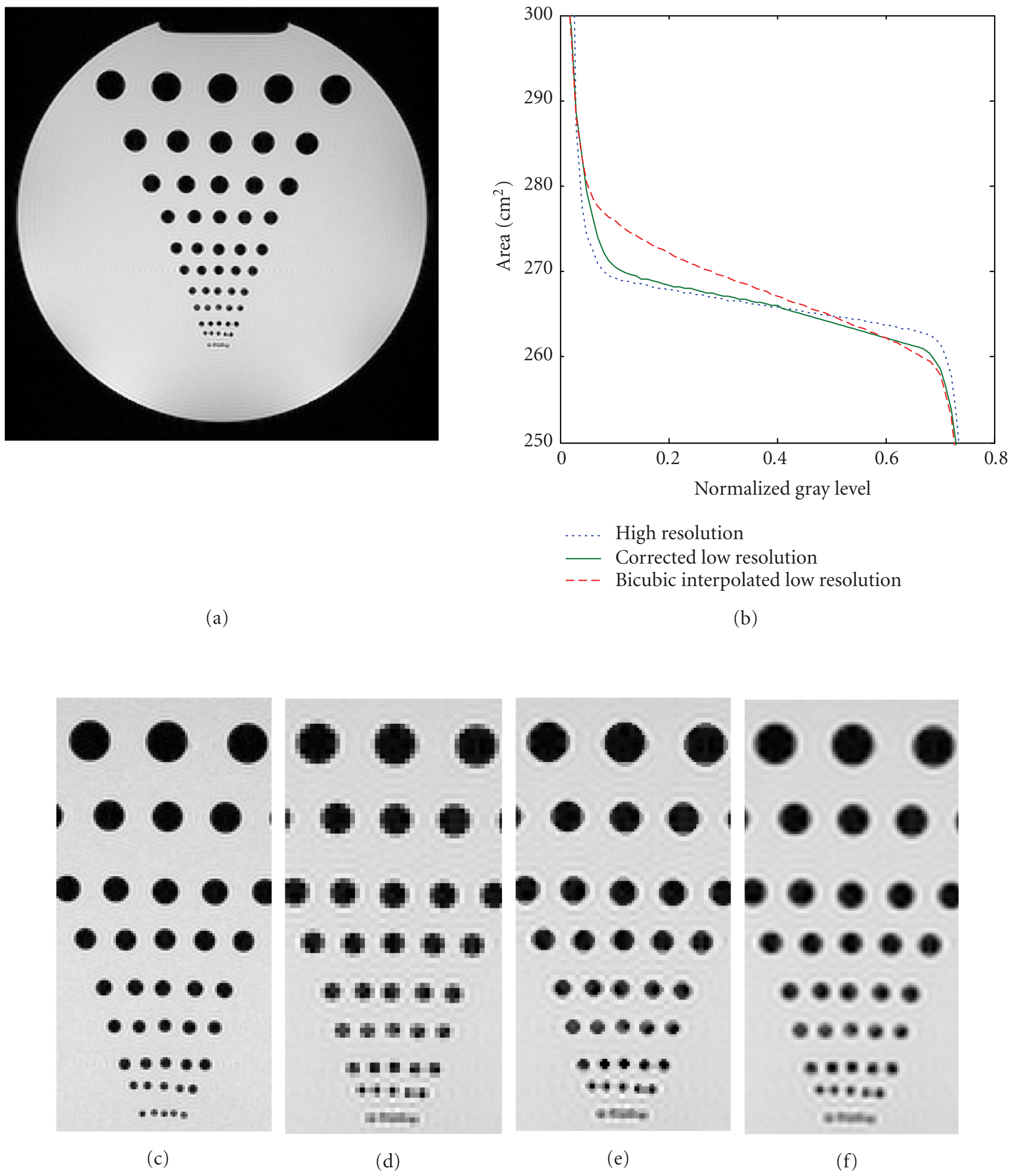

Figure 7: Test on physical MRI phantom. A phantom has been acquired with $256 \times 256$ matrix ($1 \times 1$ mm$^2$ shown in panel (d)) and corrected with the proposed method to yield a $512 \times 512$ image, shown in panel (a) and in details in panel (e). Panel (b) shows areas computed by cumulative sum of the histograms for different images displayed on the bottom row: an acquired high resolution $512 \times 512$ image at the same location (dotted line and panel (c)), the corrected image (solid line and panel (e)), and the $512 \times 512$ bicubic interpolation (dashed line and panel (f)). Area, as measured by the middle of the horizontal slope, is more accurate with the reverse diffusion than with bicubic interpolation.

The corrected image shows sharp contour of the circular plastic rods down to the 1 mm rod size. The 1.5 mm size rod was slightly improved, and the 1 mm size was almost not changed, a desirable behavior when features are equal to or lower than the voxel size ($1 \times 1$ mm). The sharpening of the edges with RD correction is more accurate than the bicubic interpolation method. The area measured by thresholding at increasing gray-level value was closer to area measured on a high-resolution image than with bicubic interpolation (Figure 7, panel (b)). This behavior is an important feature of the proposed method, and diffusion-based filters in general. Because of the flow conservation imposed in the algorithm and adiabatic conditions at image borders, the integral over the whole image is kept constant during the diffusion process. This is very important for medical images where often such integral computations are performed to quantify pathology.

### 4.4. MRI of human head and neck

Finally we tested the method on actual sagittal slice of human head. High-resolution images could be compared to corrected low-resolution images taken at the same location. Figure 8 shows one slice of this experiment, where improvement is obvious, as exemplified in the details of the pons shown in panels (b), (c), (d), and (e), respectively, for the high resolution, low resolution, corrected with RD, and interpolated with bicubic kernel images, respectively. Details on the corrected images are sharper and comparable to the high-resolution image details. Importantly no artifact can be observed: partial volume effect is reduced when possible and the image is unchanged otherwise. Figure 9 shows a PDW image of the neck acquired with surface array coils that induce strong intensity inhomogeneity. RD decreases the partial volume effect, even in these low-contrast, low-SNR images.

### 5. DISCUSSION AND CONCLUSION

The proposed method gives improved subjective image quality and truer intensities as compared to conventional interpolation schemes. The reverse diffusion method allows one to interpolate images while recovering edges which have been blurred due to the partial area/volume effect. Although improvements in qualitative image quality is not our goal, subjective blurring of interpolated images is reduced on synthetic, physical phantom, and brain MR images (Figures 5–9) as compared to conventional interpolation schemes used in medical image analysis. Importantly, these improvements are present without introducing artifacts and without adjustment of free parameters. Quantitative evaluation on the synthetic images showed that the true signal intensity was recovered at edges and that the interpolated image was very close to the actual high-resolution image (Figures 3 and 4). Very importantly, the signal is conserved (Figure 7). Because of this constraint, measurement of signal intensity should be more accurate. In addition any segmentation method based on gray level should also benefit.

So far our method has been implemented on 2D images. We do not foresee any difficulty to extend the algorithm to 3D. The potential benefit in 3D should be greater because medical images are often acquired with thick slices. Reduction of partial voluming in all three directions should help to segment convoluted or elongated anatomical structures such as brain or vessels, and should improve multiplanar reformatting and volumetric visualization methods. For example, in Figure 9 the image shown is one slice among ten taken to evaluate atherosclerosis in carotid arteries. In these thick slices (resolution is $0.5\,\text{mm} \times 0.5\,\text{mm} \times 3\,\text{mm}$), partial volume degradation may occur in the $z$-direction that is not corrected. With extension to three dimensions, the new method should address this issue. However in some situations, the distance between two slices can be higher than the slice thickness, creating gaps in the dataset. Some interpolation methods can estimate the missing data, but since RD conserves signal intensity, it is not suitable for noncontiguous image data.

RD is relatively insensitive to noise. That is, we found that partial voluming can be reduced on simulated data even in the presence of noise (Figure 4), as well as on MR images with typical noise levels (Figures 6–9). However, it should be noted that as RD enhances edges blurred by partial volume effect, it acts as a high-pass filter and enhances noise. Indeed we corrected a uniform image with noise and verified the increased energy in the high-frequency portion of the spectrum (results not shown). A preprocessing step of low-pass filtering could be used with very noisy data.

RD can recover sharp edges. Because a model for partial voluming is used in our method, infinitely sharp edges could theoretically be fully recovered to the limit of the data resolution. In contrast, features smaller than a voxel cannot be recovered since no particular information is available to the algorithm. RD can thus be considered as a nonlinear high-pass filter, acting mostly for edge information and able to estimate the high-frequency components of edges above the Nyquist frequency. The high frequencies in the original data higher than the Nyquist frequency that are not recovered correspond to structure smaller than a voxel size, as showed in results on our synthetic phantom (Figure 3). Satisfactorily in this latter case, the method does not change the data nor produces artifact.

Entropy of images is reduced by the RD process after correction. The graph of the entropy showed in Figure 3 (bottom-right panel) is typical for noise-free images. It decreases rapidly and then converges to a final lower value. This observation supports the argument put forth in the introduction section: compared to classic interpolation techniques where only the gray level of the voxels is taken into account, the proposed method uses the extra knowledge that the images have been subject to partial volume artifact. That is, the entropy of the corrected image is lower than the original low-resolution data, and lower than with bicubic or bilinear interpolations (results not shown). In the presence of noise, the curve of entropy as a function of iteration number is different: the entropy does not decrease as much as compared to the noise-free case, and rather increases if the noise level is high. Indeed, when the algorithm is run on an image with random noise only (results not shown), the entropy increases monotically at each iteration.

Reversing diffusion to enhance interpolated images has been previously proposed [8, 28], and is also closely related to shock filters [10, 11, 22, 23]. Gilboa et al. [8] add an extra term to the original anisotropic diffusion scheme such that the diffusion coefficient is negative for high gradients and positive for small ones. In order to stabilize the method, negative flow is balanced by the positive one, and the diffusion coefficient is set to zero at very high gradients, presumably where there is an edge. Their scheme called forward-backward diffusion uses two extra parameters, and produces very impressive image enhancement. The authors suggest applying it after bicubic interpolation to obtain super-resolution images. Our method is different in three fundamental aspects: there is no filtering in homogeneous regions, the integral over a voxel is kept constant, and

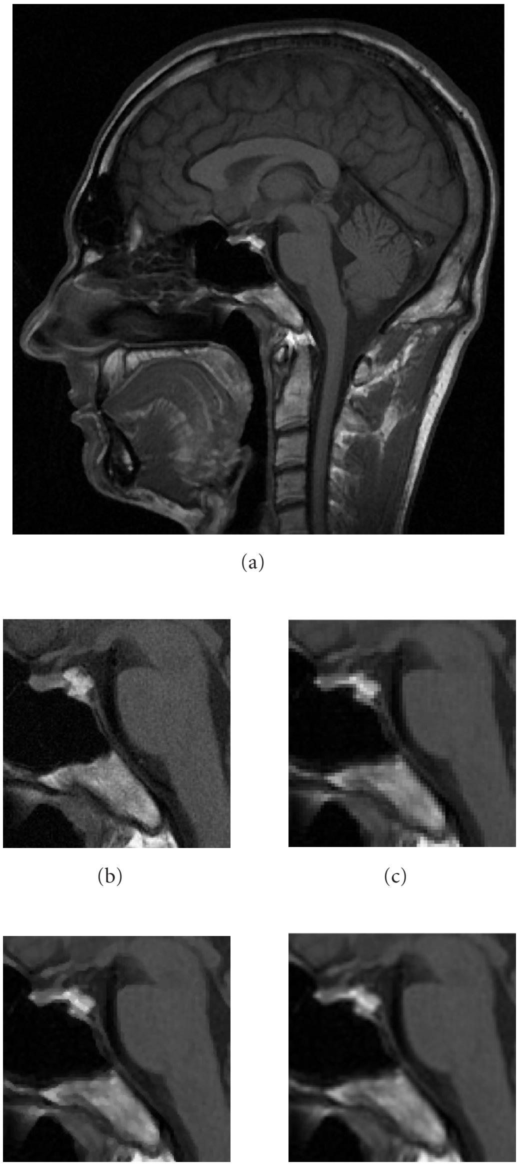

(a) (b) (c) (d) (e)

Figure 8: Test on actual human MRI. A 2D sagittal slice of a human head has been acquired using $256 \times 256$ points and corrected using the proposed method to yield a new $512 \times 512$ image (a). Details of the medulla oblongata are shown for (b) same image acquired at high resolution $512 \times 512$, (c) input $256 \times 256$ image, (d) corrected $512 \times 512$ image, and (e) bicubic interpolation of the input image.

no extra parameter is needed to characterize an edge. As a result, images processed with RD are very close to true data (Figures 3, 4, 7, and 8), but they can be noisier than those obtained using those alternative methods. We are investigating ways to extend our method to include noise reduction.

In our current implementation the point spread function should be small relative to a voxel. One of our main assumptions, shared by almost all methods correcting partial volume effect [16], is that the signal is averaged over a voxel. This requires the point spread function (PSF) to be about equal or smaller than the size of a voxel at the coarse resolution, which is reasonable in MRI. If this is not the case, most of the blurring would come from the PSF convolution with the data rather than signal averaging inside the voxel.

For the case of a large PSF, it would be necessary to control the flow of material over a spatial support corresponding to the size of the PSF rather than the original voxel. In this event, reverse diffusion could be considered as a deconvolution method. The heat conduction equation has a Gaussian distribution as a solution. An explicit numerical implementation of the diffusion equation is equivalent to filtering by convolving with a Gaussian whose standard deviation is the square root of twice the diffusion time [29]. However, a more realistic deconvolution algorithm should take into account

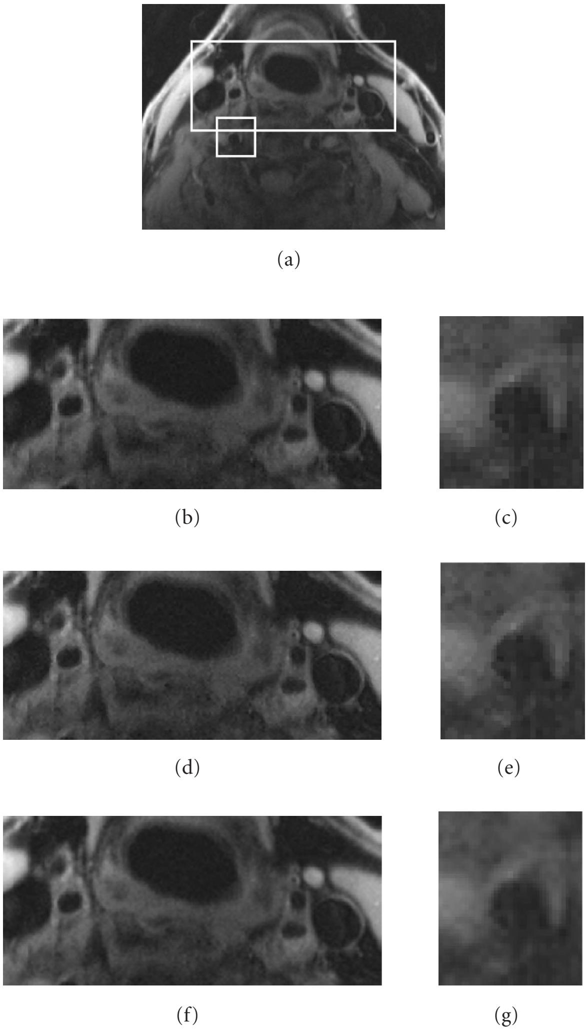

FIGURE 9: Low-contrast image interpolation. A PDW MR image of a patient neck suffering from atherosclerosis in the left internal carotid artery is shown in (a) along with two details in panels (b) and (c). The same details are shown in (d) and (e) after RD interpolation, and in (f) and (g) after bicubic interpolation. Even in this low-SNR and low-contrast example, partial volume has been reduced as seen in the artery boundaries for example.

the size of the deconvolution kernel and should track the spatial spread of the signal for each voxel.

In conclusion, we have presented a totally automatic method to reduce partial volume effect of images. It does not need any parameters and restores images by implementing the diffusion equation backward in time. As compared to conventional interpolation techniques, we obtained excellent results on simulated multiclass images, gray-level facial and text images, and MRI scans.

### ACKNOWLEDGMENTS

This work was supported by Ohio Wright Center of Innovation and Biomedical Research and Technology Transfer award: "The Biomedical Structure, Functional and Molecular Imaging Enterprise." We are grateful to Professors Daniela Calvetti, Jiayang Sun, and Gerald Saidel for insightful discussions.

### REFERENCES

[1] J. G. Proakis and D. G. Manolakis, *Digital Signal Processing: Principles, Algorithms and Applications*, Prentice Hall, Upper Saddle River, NJ, USA, 3rd edition, 1995.

[2] T. M. Lehmann, C. Gonner, and K. Spitzer, "Survey: interpolation methods in medical image processing," *IEEE Transactions on Medical Imaging*, vol. 18, no. 11, pp. 1049–1075, 1999.

[3] P. Thevenaz, T. Blu, and M. Unser, "Interpolation revisited [medical images application]," *IEEE Transactions on Medical Imaging*, vol. 19, no. 7, pp. 739–758, 2000.

[4] G. J. Grevera and J. K. Udupa, "Shape-based interpolation of multidimensional grey-level images," *IEEE Transactions on Medical Imaging*, vol. 15, no. 6, pp. 881–892, 1996.

[5] H.-C. Ting and H.-M. Hang, "Edge preserving interpolation of digital images using fuzzy inference," *Journal of Visual Communication and Image Representation*, vol. 8, no. 4, pp. 338–355, 1997.

[6] S. Battiato, G. Gallo, M. Mancuso, G. Messina, and F. Stanco, "Analysis and characterization of super-resolution reconstruction methods," in *Sensors and Camera Systems for Scientific, Industrial, and Digital Photography Applications IV*, M. M. Blouke, N. Sampat, and R. J. Motta, Eds., vol. 5017 of *Proceedings of SPIE*, pp. 323–331, Santa Clara, Calif, USA, January 2003.

[7] S. Morigi and F. Sgallari, "3D long bone reconstruction based on level sets," *Computerized Medical Imaging and Graphics*, vol. 28, no. 7, pp. 377–390, 2004.

[8] G. Gilboa, N. Sochen, and Y. Y. Zeevi, "Forward-and-backward diffusion processes for adaptive image enhancement and denoising," *IEEE Transactions on Image Processing*, vol. 11, no. 7, pp. 689–703, 2002.

[9] I. Pollak, A. S. Willsky, and H. Krim, "Image segmentation and edge enhancement with stabilized inverse diffusion equations," *IEEE Transactions on Image Processing*, vol. 9, no. 2, pp. 256–266, 2000.

[10] L. Alvarez and L. Mazorra, "Signal and image-restoration using shock filters and anisotropic diffusion," *SIAM Journal on Numerical Analysis*, vol. 31, no. 2, pp. 590–605, 1994.

[11] L. Remaki and M. Cheriet, "Numerical schemes of shock filter models for image enhancement and restoration," *Journal of Mathematical Imaging and Vision*, vol. 18, no. 2, pp. 129–143, 2003.

[12] S. Ruan, C. Jaggi, J. Xue, J. Fadili, and D. Bloyet, "Brain tissue classification of magnetic resonance images using partial volume modeling," *IEEE Transactions on Medical Imaging*, vol. 19, no. 12, pp. 1179–1187, 2000.

[13] P. Santago and H. D. Gage, "Statistical models of partial volume effect," *IEEE Transactions on Image Processing*, vol. 4, no. 11, pp. 1531–1540, 1995.

[14] D. H. Laidlaw, K. W. Fleischer, and A. H. Barr, "Partial-volume Bayesian classification of material mixtures in MR volume data using voxel histograms," *IEEE Transactions on Medical Imaging*, vol. 17, no. 1, pp. 74–86, 1998.

[15] H. S. Choi, D. R. Haynor, and Y. Kim, "Partial volume tissue classification of multichannel magnetic resonance images—a mixel model," *IEEE Transactions on Medical Imaging*, vol. 10, no. 3, pp. 395–407, 1991.

[16] K. Van Leemput, F. Maes, D. Vandermeulen, and P. Suetens, "A unifying framework for partial volume segmentation of brain MR images," *IEEE Transactions on Medical Imaging*, vol. 22, no. 1, pp. 105–119, 2003.

[17] A. Ismail-Zadeh, G. Schubert, I. Tsepelev, and A. Korotkii, "Inverse problem of thermal convection: numerical approach and application to mantle plume restoration," *Physics of the Earth and Planetary Interiors*, vol. 145, no. 1–4, pp. 99–114, 2004.

[18] K. A. Ames, G. W. Clark, J. F. Epperson, and S. F. Oppenheimer, "A comparison of regularizations for an ill-posed problem," *Mathematics of Computation*, vol. 67, no. 224, pp. 1451–1471, 1998.

[19] J. M. Marbán and C. Palencia, "A new numerical method for backward parabolic problems in the maximum-norm setting," *SIAM Journal on Numerical Analysis*, vol. 40, no. 4, pp. 1405–1420, 2002.

[20] A. Hasanov and J. L. Mueller, "A numerical method for backward parabolic problems with non-selfadjoint elliptic operators," *Applied Numerical Mathematics*, vol. 37, no. 1-2, pp. 55–78, 2001.

[21] P. Perona and J. Malik, "Scale-space and edge detection using anisotropic diffusion," *IEEE Transactions on Pattern Analysis and Machine Intelligence*, vol. 12, no. 7, pp. 629–639, 1990.

[22] S. Osher and L. I. Rudin, "Feature-oriented image enhancement using shock filters," *SIAM Journal on Numerical Analysis*, vol. 27, no. 4, pp. 919–940, 1990.

[23] M. Breuss, T. Brox, T. Sonar, and J. Weickert, "Stabilised nonlinear inverse diffusion for approximating hyperbolic PDEs," in *Proceedings of the 5th International Conference on Scale Space and PDE Methods in Computer Vision (Scale Space '05)*, vol. 3459 of *Lecture Notes in Computer Science*, pp. 536–547, Hofgeismar, Germany, April 2005.

[24] K. W. Morton and D. F. Mayers, *Numerical Solution of Partial Differential Equations*, Cambridge University Press, Cambridge, UK, 1994.

[25] F. Catté, P.-L. Lions, J.-M. Morel, and T. Coll, "Image selective smoothing and edge detection by nonlinear diffusion," *SIAM Journal on Numerical Analysis*, vol. 29, no. 1, pp. 182–193, 1992.

[26] G. P. Penney, J. A. Schnabel, D. Rueckert, M. A. Viergever, and W. J. Niessen, "Registration-based interpolation," *IEEE Transactions on Medical Imaging*, vol. 23, no. 7, pp. 922–926, 2004.

[27] O. Salvado and D. L. Wilson, "Entropy based method to correct intensity inhomogeneity in MR images," in *Proceedings of 26th Annual international Conference of IEEE Engineering in Medicine and Biology Society (EMBS '04)*, vol. 1, pp. 164–167, San Francisco, Calif, USA, September 2004.

[28] N. Sochen and Y. Y. Zeevi, "Super-resolution of grey-level images by inverse diffusion processes," in *Proceedings of 9th Mediterranean Electrotechnical Conference (MELECON '98)*, vol. 1, pp. 91–95, Tel-Aviv, Israel, May 1998.

[29] T. Lindeberg, "Scale-space for discrete signals," *IEEE Transactions on Pattern Analysis and Machine Intelligence*, vol. 12, no. 3, pp. 234–254, 1990.

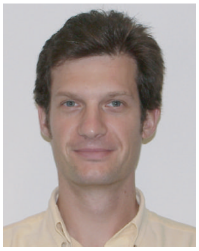

**Olivier Salvado** received his M.S. degree in electrical engineering from ESIEE, Marne-la-Vallée, France, in 1994, and the M.S. degree in modelisation from the University Paris XII, Creteil, France in 1994. He worked for seven years for Voest Alpine International Inc. an international engineering company. His last position was Principal Engineer, and he was Technical Leader in system control design. He holds two patents. Currently he is pursuing the Ph.D. degree in biomedical engineering at Case Western Reserve University. His research interests include digital image processing, magnetic resonance imaging, and atherosclerosis diagnostic. In particular he is working on PDE methods to filter and reduce partial volume effect, registration artifacts, intensity inhomogeneity correction, and tissue classification.

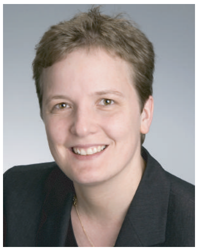

**Claudia M. Hillenbrand** received her M.S. and Ph.D. degrees in physics from the Julius-Maximilians University of Wuerzburg, Germany. She joined Case Western Reserve University as a Postdoctoral Fellow in 2000, and became an Assistant Professor of Radiology at University Hospitals of Cleveland in 2003. In 2005 she joined the faculty of St. Jude Children's Research Hospital in Memphis, Tenn, while still holding an adjunct faculty position at Case Western Reserve University. Her field of expertise is in magnetic resonance imaging physics. Her research activities include MR guided interventions, MR sequence development, and the MR assessment of disease or therapeutically induced vasculopathy.

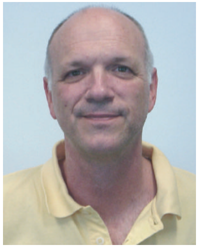

**David L. Wilson** received his Ph.D. degree in electrical engineering from Rice University, Houston, Tex. He is now professor of Biomedical Engineering and Radiology at Case Western Reserve University. He has interests in image processing, registration, and cellular and molecular imaging. He has a significant track record of federal research funding, over 65 refereed journal publications, and 7 patents. He has been active in the development of international conferences, including the IEEE EMBS and IEEE ISBI conferences. He has actively developed biomedical imaging at Case. He has led the BME faculty recruitment effort in imaging, and he has served as PI or has been an active leader on multiple research and equipment developmental awards to Case. Currently, he is the Case PI on the Ohio State University, Wright Center of Innovation Award, The Biomedical Structural, Functional and Molecular Imaging Enterprise, an investment of > $17 M by the state of Ohio, which includes local industries such as Philips Medical Systems. Finally, he serves as Associate Chair in the Department of Biomedical Engineering.

